# Edge detection networks inspired by neural mechanisms of selective attention in biological visual cortex

**DOI:** 10.3389/fnins.2022.1073484

**Published:** 2022-11-22

**Authors:** Zhenguang Zhang, Chuan Lin, Yakun Qiao, Yongcai Pan

**Affiliations:** School of Automation, Guangxi University of Science and Technology, Liuzhou, China

**Keywords:** edge detection, biological vision, visual cortex, self-attention mechanism, convolutional neural network

## Abstract

Edge detection is of great importance to the middle and high-level vision task in computer vision, and it is useful to improve its performance. This paper is different from previous edge detection methods designed only for decoding networks. We propose a new edge detection network composed of modulation coding network and decoding network. Among them, modulation coding network is the combination of modulation enhancement network and coding network designed by using the self-attention mechanism in Transformer, which is inspired by the selective attention mechanism of V1, V2, and V4 in biological vision. The modulation enhancement network effectively enhances the feature extraction ability of the encoding network, realizes the selective extraction of the global features of the input image, and improves the performance of the entire model. In addition, we designed a new decoding network based on the function of integrating feature information in the IT layer of the biological vision system. Unlike previous decoding networks, it combines top-down decoding and bottom-up decoding, uses down-sampling decoding to extract more features, and then achieves better performance by fusing up-sampling decoding features. We evaluated the proposed method experimentally on multiple publicly available datasets BSDS500, NYUD-V2, and barcelona images for perceptual edge detection (BIPED). Among them, the best performance is achieved on the NYUD and BIPED datasets, and the second result is achieved on the BSDS500. Experimental results show that this method is highly competitive among all methods.

## Introduction

Edge detection is one of the most fundamental tasks in computer vision (Canny, 1986). Its main purpose is to extract the object boundary and prominent edge containing the main information of the target from the natural image, ignoring other unimportant details in the image. The edge and boundary of objects are important in other middle and advanced computer vision tasks, which help to improve their performance. Such as target recognition and detection (Ferrari et al., 2007; Liu et al., 2020), image segmentation (Arbelaez et al., 2010; Muthukrishnan and Radha, 2012), semantic segmentation (Lin et al., 2017), and other visual tasks. Therefore, edge detection is also a research hotspot in the field of computer vision.

With the increasing demand for other visual tasks and the improvement of performance requirements for basic tasks, edge detection methods are constantly developing and improving. Early edge detection methods such as Prewitt (1970), Duda and Hart (2003), Canny (1986) mainly extracted edges by calculating local gradient changes of images. Later, in order to obtain better edge detection performance than traditional methods. Some researchers proposed a detection method that mimics biological vision based on the effective biological vision mechanism for edge detection in the biological vision system. For example, Grigorescu et al. (2003) proposed isotropic and anisotropic methods, while other researchers proposed CO (Color-opponent) (Yang et al., 2015b), SCO (Double-Opponency and Spatial Sparseness Constraint) (Yang et al., 2015a), etc., which achieved better performance than traditional edge detection methods. In order to improve the performance of edge detection, some experts and scholars use machine learning to process the edge detection task as a pixel-level binary classification task. An edge detection method based on unsupervised learning is proposed to better integrate the whole image and local information. For example, the Pb algorithm proposed by Martin et al. (2004), gPb algorithm proposed by Arbelaez et al. (2010), and oriented edge forests (OEF) algorithm proposed by Hallman and Fowlkes (2015).

With the development of deep learning in recent years, the performance of edge detection has been greatly improved. HED (Holistically-nested edge detection) is the earliest edge detection method based on CNN (Convolutional Neural Network). It is also the first end-to-end edge detection model proposed by Xie and Tu (2015) inspired by fully convolutional networks (FCN) (Long et al., 2015), which achieves the most advanced performance. Later, Liu et al. (2017) proposed RCF (Richer convolutional features) based on HED, Wang et al. (2017) proposed CED (Crisp edge detector), and other researchers proposed more edge detection methods based on CNN (Deng et al., 2018; He et al., 2019; Cao et al., 2020; Deng and Liu, 2020; Lin et al., 2020), all of which achieved advanced performance. On the NYUD-v2 data set (Silberman et al., 2012), the detection performance has been boosted from 0.632 (Arbelaez et al., 2010) to 0.765 (He et al., 2019) in optimal dataset scale (ODS) F-measure.

As mentioned above, the edge detection method based on CNN has achieved impressive results. But there are still some problems worth studying. First, they ignore the important impact that coding networks can have. Edge detection methods based on CNN generally adopt codec network structure (Xie and Tu, 2015; Liu et al., 2017; Wang et al., 2017). The encoding network mainly extracts feature information and the decoding network integrates feature information. Researchers usually directly use deep convolutional neural network VGG16 (Simonyan and Zisserman, 2015) or ResNet (He et al., 2016) as the encoding network and design the decoding network with a complex structure to obtain good performance. Second, researchers (Xie and Tu, 2015; Liu et al., 2017; Wang et al., 2017; He et al., 2019; Lin et al., 2020) usually fuse the output of the coding network in a top-down manner when designing the decoding network. In the research, they pay more attention to the method of recovering the feature map and pay less attention to other methods that can integrate the whole feature. So why not consider other ways to improve model performance besides decoding networks? Why can’t bottom-up fusion be used in decoding networks or top-down fusion be combined with bottom-up fusion?

With the gradual deepening of the research on edge detection, some experts and scholars have found that it is difficult to significantly improve the overall performance of the model only for the design of decoding network (Deng et al., 2018; Cao et al., 2020; Deng and Liu, 2020). To this end, they began to explore other ways to improve edge detection performance. Deng et al. (2018) obtained finer edges by designing new loss functions. Cao et al. (2020) improved the overall performance of the model by designing a new decoding network and combining the improved loss function. Later, Deng and Liu (2020) proposed to add a super-convolution module between the coding network and the decoding network to deal with the output of the encoding network at a deeper level and to combine the improved loss function to improve the overall performance of the model. Wibisono and Hang (2020a) proposed a fast inference network for edge detection using expanded convolution to design backbone networks. Su et al. (2021) proposed a new edge detection Network PiDiNet (Pixel Difference Network) by combining difference operator and convolution operation.

**FIGURE 1:**
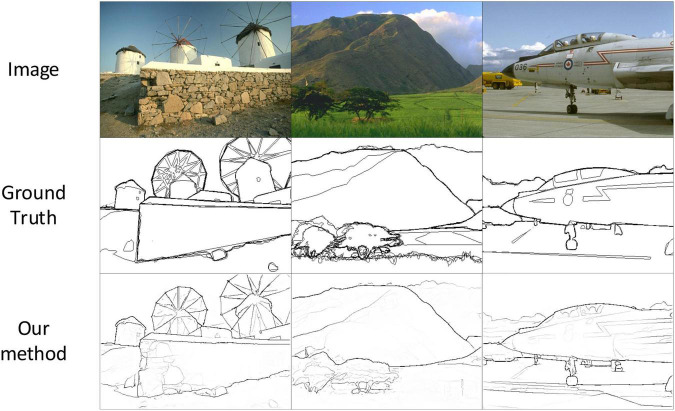
Partial test results of our method on the BSDS500 dataset. Source photos: BSDS500 dataset - publicly available dataset.

Based on the above analysis, in view of some problems existing in the current methods, and combined with the connection between neural network and Biological Vision System (Fukushima et al., 1988; Hao et al., 2021). In this paper, we proposed a new edge detection model (MEDNet, Modulation encoding and decoding network) inspired by the selective mechanism in biological visual pathways V1, V2, and V4 (Yoshioka et al., 1996; Luck et al., 1997; Marcus and Essen, 2002; Allen et al., 2018). Figure 1 shows the partial test results of MEDNet on the BSDS500 dataset. In the new edge detection model, we use VGG16 (Simonyan and Zisserman, 2015) as the encoding network, which is the same as the previous method. Inspired by the spatial selectivity mechanism in V1, V2, and V4, we use the self-attention mechanism in Transformer (Dosovitskiy et al., 2020; Liu et al., 2021; Wang et al., 2021) to design a modulation enhanced network (MENet). A new encoding network, modulation coding network (MCNet), is formed by combining the modulation enhancement network and encoding network, which enhances the feature extraction ability of the encoding network. The overall performance of the model is further improved by enhancing the feature extraction capability of the encoding network. In addition, based on the functional structure of the bio-visual Pathway (Okazawa et al., 2016) and CNN, and combining the characteristics of V1, V2, V4, and the modulation encoding network, the modulation encoding network is reasonably corresponding to V1, V2, V4 in the bio-visual pathway. In addition, in view of the problem that the previous decoding network only adopts the top-down fusion method to achieve feature integration. In this paper, based on the main function of the IT area in the biological vision system (Bear et al., 2020) (responsible for recognizing objects and integrating feature information), we design a new decoding network–Double Decoding Network (DDNet). We decode the output of the coding network from top to bottom and from bottom to top, respectively, and the decoding fusion process does not interfere with each other. Finally, we fuse the output of the two decoding networks to obtain the final edge output and achieve better performance.

In the rest of the paper, we introduce some previous work in related fields and describe in detail the methods we propose in this paper. In addition, for the method proposed in this paper, detailed experimental analysis is carried out on three publicly available data sets BSDS500 (Arbelaez et al., 2010), NYUD-V2 (Silberman et al., 2012), and barcelona images for perceptual edge detection (BIPED) (Poma et al., 2019). By comparing the experimental results with those of other edge detection methods, our method has proven to be competitive. Finally, we summarize the whole paper.

## Related work

In this part, we introduce the traditional edge detection method, the edge detection method based on biological vision, and the edge detection method based on CNN. Among them, this paper mainly involves the biomimetic vision method and the method based on CNN.

Traditional edge detection method. The traditional edge detection method mainly extracts the target edge from the image by the local grayscale, color, texture, and background change. Such as Prewitt (1970), Canny (1986), Duda and Hart (2003). Although these methods can achieve the extraction of target edges, they only focus on the change of local information in the extraction process, which leads to the problems of poor continuity and low accuracy of extracted edges. And the traditional edge detection method is susceptible to the interference of background and texture noise in the process of image edge extraction, which also leads to the extracted edge still containing a lot of background and texture information. Hence, conventional edge detection methods can hardly meet the requirements of other visual tasks.

An edge detection model for biomimetic vision. In order to improve the performance of edge detection methods and better meet the requirements of middle and advanced visual tasks, some experts and scholars began to explore new edge detection methods. Inspired by Hubel and Wiesel (1962) discovery that primary visual cortex (V1) neurons have the function of detecting edges and lines, some researchers began to study the role of biological vision in image edge extraction. Then they proposed a series of edge detection methods that mimic biological vision. For example, the edge detection model simulates the inhibition of simple cell and complex cell nCRF (non-Classical Receptive Field) to CRF (Classical Receptive Field) response in the biological vision system (Grigorescu et al., 2003). The suppression effect of nCRF on CRF response is simulated by using the DoG operator, and the suppression of texture and background in the image is realized. Based on the color antagonism mechanism from the retina to visual cortex (V1) and spatial sparsity constraint strategy (SSC), the SCO edge detection model was proposed by Yang et al. (2015a). Later, Tang et al. (2019) combined the modulation of CRF by nCRF in biological vision with deep learning, and proposed a learnable edge detection model inspired by biological vision, which achieved the best performance in the edge detection method of biological vision.

Edge detection method based on CNN. In recent years, CNN has made impressive achievements in the field of edge detection. Xie and Tu (2015) proposed the first end to end edge detection model HED based on CNN, which achieved the most advanced performance in the field of edge detection. In HED they retained the 13 convolutional layers of VGG16 as the coding network and divided it into five parts for output according to the modified structure. Then the final edge is obtained by integrating ve different resolution outputs of the coding network through the decoding network. Inspired by FCN, Yang et al. (2016) proposed the full convolutional codec network CEDN. CEDN uses VGG16 as the encoding network and the input can be any size image. After feature extraction in coding network, deconvolution and anti-pooling layer are used to restore the size of feature map in decoding network. Maninis et al. (2016) proposed an edge detection algorithm COB with convolution-oriented boundary structure. It achieves better detection performance by fusing multi-scale contour information. Liu et al. (2017) improved on HED and proposed an edge detection network RCF with richer features. In RCF, Liu et al. (2017) used the same coding network and the same partition as HED, but the difference was that they output the results of all 13 convolutional layers in the coding network. In addition, Liu et al. introduced multi-scale results into RCF, which improved the performance of the model. After that, Wang et al. (2017) improved the decoding network and adopted sub-pixel convolution layer by layer to sample and fuse the output of the coding network in the decoding network, and proposed a network CED that could obtain finer edges and achieve good performance. Other researchers have also proposed edge detection models based on CNN, such as BDCN proposed by He et al. (2019), which achieved good performance by using two-way cascaded networks. DRC proposed by Cao et al. (2020) improved the overall performance of the model by designing fusion modules and improving loss functions. The DSCD (deep structure contour detection) proposed by Deng and Liu (2020) improves the overall performance of the model by adding hyper convolutional modules between the encoding network and the decoding network and designing new loss functions. Poma et al. (2019) put forward Dense Extreme Incident Network for Edge Detection (Dexined), which can achieve excellent performance without pre-training. It can also be applied to multiple datasets without fine-tuning and has great generalization performance. Deng et al. (2021) designed the coding network combined with MobileNetV2 to extract features, and used the new decoding network to compress features. Finally, combined with the designed loss function, a modern edge detection model was proposed. Su et al. (2021) proposed a (PiDiNet) for edge detection by combining the difference operator in traditional operators with convolution operation, and achieved excellent performance in detection efficiency and parameters. Wibisono and Hang (2020a) designed a backbone network with dilated convolutions and proposed FINED for edge detection. TIN2 (Wibisono and Hang, 2020b) is designed in conjunction with the traditional contour detection step, and both achieve excellent performance in terms of detection efficiency. Recently, Transformer has attracted the attention of a large number of researchers by demonstrating strong performance by achieving the best results in various computer vision tasks. Pu et al. (2022) applied it to the field of edge detection and proposed a new edge detector EDTER based on Transformer, which achieved the best results. EDTER first uses a global transformer encoder to extract global contextual information and then a local transformer encoder to extract local features. At the same time, global information and local features are used to obtain clearer boundaries and significant edges, thus achieving better performance.

To sum up, it can be found that although traditional edge detection methods can achieve the extraction of target image edges, their accuracy is difficult to meet the requirements of other visual tasks. Although the method of biomimetic vision achieves the suppression of image texture and background to a certain extent. However, most of the current edge detection methods of biomimetic vision use mathematical formulas to simulate some characteristics or mechanisms in the biological vision system. There are still some limitations. The edge detection method based on CNN has achieved the most advanced performance, but the researchers pay more attention to the design of the decoding network. Given the problems existing in the current methods, inspired by the previous biomimetic vision model and combined with the selectivity mechanism in the biological visual pathways V1, V2, and V4. We propose a new modulation enhancement network. The overall performance of the model is improved by enhancing the feature extraction capability of the coding network. At the same time, we designed a new decoding network to improve the performance of the model.

## Edge detection network structure combining convolutional neural network and biological vision

### Biological visual mechanism

Selective mechanisms in biological vision. In the biological vision system, the information transmission starts from the retina. The visual information is processed and transformed by the retina and transmitted to Lateral geniculate nucleus (LGN) by ganglion cells, and then projected to primary visual cortex V1 after LGN processing, which carries out preliminary processing and feature extraction for LGN-processed visual information. The transmission of visual information from the retina to the LGN to the V1 region is known as the first visual pathway (Mishkin et al., 1983; Bear et al., 2020). The information processed in area V1 then travels through two parallel pathways to different brain regions for different functions. The processing and transmission of V1→V2→V4→IT [Inferior temporal (IT) cortex] is called the ventral pathway, also known as the “What” pathway (Ungerleider and Haxby, 1994). It is sensitive to color, shape, and direction in visual information (“→” indicates the direction of information transmission). The processing transmission of the V1→V2→V3→MT (Middle temporal) cortex is called the dorsal pathway, also known as the “Where” pathway (Ungerleider and Haxby, 1994). It is more sensitive to motor information in visual information. For the edge detection task, feature extraction is mainly based on color, shape, and other information in the image. Therefore, we focused on information processing transmission in the ventral pathway.

As scientists continue to explore the brain, some researchers have discovered the existence of selective mechanisms in biological vision (Yoshioka et al., 1996; Luck et al., 1997; Allen et al., 2018). Physiological studies have also shown that V1, V2, and V4 in biological visual pathways have selective mechanisms in information processing and extraction. That is, they have different processes for different information, they will be more sensitive to some important information, and ignore some unimportant details. Based on and inspired by the existence of this selectivity in V1, V2, V4. In this paper, we use the self-attention mechanism (Wang et al., 2021) in Transformer to design the MENet, which realizes the selective extraction of feature information according to the global information of the image. Then a new coding network, MCNet, was formed by combining MENet with the coding network. At the same time, based on the structure of CNN and the connection between neural network and biological vision, the new coding network also formed a reasonable correspondence with V1, V2, and V4 in the ventral pathway both functionally and structurally.

Inferior temporal area in biological vision. The IT area is the terminus of the ventral pathway in the biological visual pathway. Its main function is to integrate the received characteristic information and identify objects (Gross et al., 1972; Tanaka, 1996; Bear et al., 2020). Neurophysiological studies have shown that neurons in the IT region have a large receptive field and are sensitive to receiving characteristic information such as color and shape. When the IT zone is damaged, it will directly affect the ability to recognize objects (Tanaka, 1996). In this paper, we design a new decoding network–double decoding network based on the feature integration function of IT area and its sensitive characteristics of color, shape and other information. Through the design of double decoding network, we realize the feature information from the top-down fusion, from the bottom-up fusion, fully extract the modulation coding network output feature information, so that the overall performance of the model has been improved. At the same time, it also forms a reasonable correspondence with the IT area in terms of function.

### Overall network structure

**FIGURE 2:**
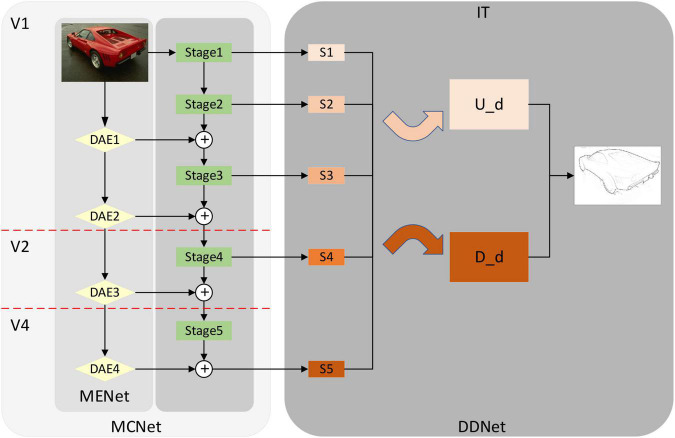
Overall network structure diagram. From left to right, modulation coding network (MCNet), dual decoding network (DDNet). Among them, MCNet also includes modulation-enhancement network (MENet) (Yellow diamond composition) and coding network (Green rectangle). Down-sampling attention enhancement module (DAE) in MENet is a down-sampling attention-enhancing module designed by using a self-attention mechanism. MCNet corresponds to V1, V2, and V4 in the biological visual pathway. The dual-decoding network DDNet integrates the feature information of up-sampling and down-sampling, respectively and outputs the feature information after integration. DDNet corresponds to the IT layer in the visual pathway. In the following sections, we describe the detailed structure of MCNet, MENet, DAE, and DDNet. Source photos: BSDS500 dataset - publicly available dataset.

Figure 2 shows the overall structure of the edge detection model proposed by us. It consists of two parts, one is the MCNet which is composed of the MENet and the coding network. The other part is the DDNet proposed in this paper. Modulation enhancement network in modulation coding network is a new network structure inspired by the selective mechanism in biological visual pathways V1, V2, and V4. By combining with the coding network, modulates the coding network and enhances the feature extraction ability of the coding network. Modulation coding network also forms a more reasonable correspondence with V1, V2, V4 in the biological visual pathway in terms of function and structure. DDNet is a dual decoding network proposed by us. It uses two decoding networks and uses different feature fusion methods to fuse the output feature information of the encoding network, which improves the performance of the model. In addition, according to the function of the IT layer in the visual pathway, which is mainly responsible for integrating feature information (Bear et al., 2020), we have formed a correspondence between decoding network and IT.

### Encoded network structure (modulation coding network and modulation enhanced network)

Modulation coding network in Figure 2 shows the modulation coding network structure diagram after we combine the modulation enhanced network and encoding network VGG16. The green part is the modified VGG16, same as the previous method (Xie and Tu, 2015; Liu et al., 2017; Wang et al., 2017; Deng and Liu, 2020). We modify VGG16, delete the full connection layer and the last pooling layer, and divide it into Stage 1, Stage 2, Stage 3, Stage 4, and Stage 5 layers according to the pooling layer. Then we combined the different outputs in MENet with the coding network according to the resolution and realized the modulation of the coding network so that more feature information was added to the coding network, especially the global feature information processed by the self-attention mechanism. The feature extraction capability of the coding network is improved, and the overall performance of the model is also improved.

Based on the structure and function of CNN and the connection between CNN and biological vision (Fukushima et al., 1988; Hao et al., 2021), we usually consider that the coding network corresponds to V1, V2, and V4 in the visual pathway. In the biological visual pathway (Bear et al., 2020), V1 mainly receives input from LGN, carries out preliminary integrated processing on the received information, and extracts primary features. After that, V1 will transfer the processed information to V2 for additional processing and feature extraction in the V2 region, and the processed information will be transferred to the V4 region. Finally, the extracted feature information will be integrated and output by the IT layer. In the coding network, the feature information extraction is the same as the step-by-step extraction in V1, V2, and V4 (Okazawa et al., 2016), which achieves the step-by-step extraction of feature information through the convolutional layer and pooling layer. In addition, considering the selective mechanism in V1, V2, and V4, we combine the modulation enhancement network based on the self-attention mechanism with the encoding network to form a new encoding network, the MCNet. The MCNet has a more reasonable correspondence with V1, V2, and V4, both functionally and structurally.

**FIGURE 3:**
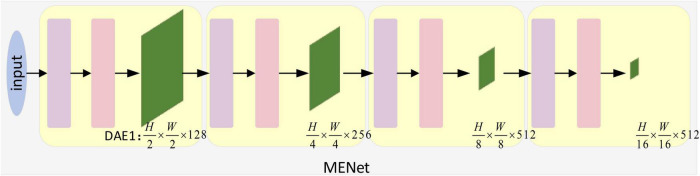
Modulation enhancement network structure diagram. Inspired by the selective mechanism in V1, V2, and V4, we design the modulation enhanced network, which can better correspond with V1, V2, and V4 by combining with the coding network. H and W represent the size of the image and feature graph, respectively, and 3, 128, 256, 512 represent the number of channels, respectively. The down-sampling embedding (DE) and transformer_encoder (TE) modules are described in detail in Figure 4.

**FIGURE 4:**
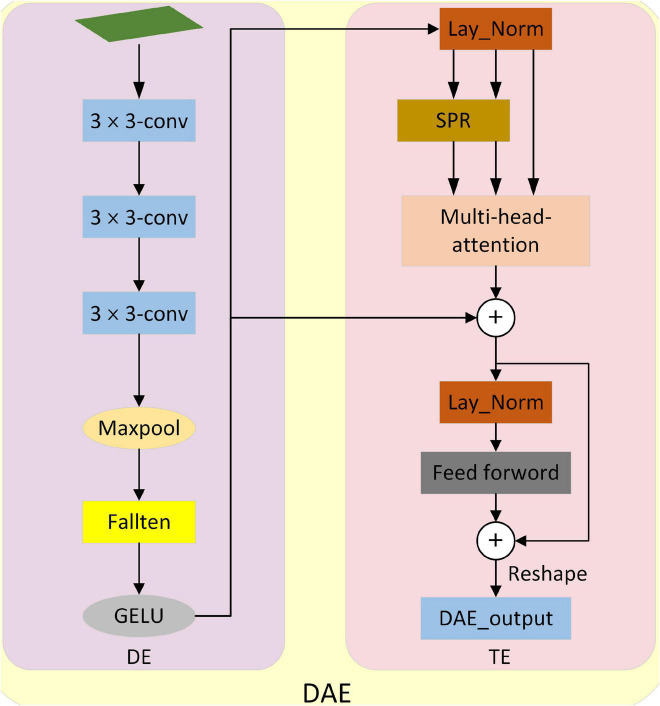
Structure Diagram of down-sampling attentional enhancement module. It consists of down-sampling embedding (DE) and transformer_encoder (TE). DE implements down-sampling processing and patch embedding operation of the input image. The TE implements selective feature extraction and outputs the results at last.

Neurophysiological studies have shown that there are selective mechanisms in biological visual pathways V1, V2, and V4 (Yoshioka et al., 1996; Luck et al., 1997). Inspired by this, in this paper, we use the self-attention mechanism of Pyramid Vision Transformer (PVT) (Wang et al., 2021) to design the modulation enhanced network MENet as shown in Figure 3, which realizes the selective extraction of global feature information in the image. Unlike most models that utilize the mechanism of self-attention, we do not block image processing and position coding in MENet but implement image patch embedding by designing a DE (Down-sampling Embedding) module. And mapping it to a vector, and realizing the down-sampling processing of the input image. Its structure is shown in Figure 4. The results of the DE module were then input to the same self-attention mechanism module as PVT (Wang et al., 2021) for re-processing, and the results were output through Layer.

Normalization and Feedforward. Finally, we restore it to its reduced size. In addition, in MENet, we combined the feature pyramid structure and used the down-sampling enhancement module to output four outputs of different scales after self-attention processing. And combine it with a coding network. By combining with coding network, the feature extraction ability of coding network is enhanced and a new coding network MCNet is formed.

Down-sampling attention enhancement module (DAE). As shown in Figure 4, the attention-enhancement module for down-sampling is proposed. The input image is first sampled by DE (Down-sampling embedding) in DAE and mapped to a vector. Then the global information of the image is extracted selectively by TE (Transformer_encoder), and the original size is restored by Reshape. Refer to the design in Wang et al. (2021) for Spatial Reduction (SPR).

Down-sampling embedding. It consists of three convolution layers, a maximum pooling layer, a fallten layer, and the activation function GELU. Where the size of the convolution layer is 3 × 3. It realizes the down-sampling processing and mapping of the input image so that the input image is mapped into a vector that can be processed by TE. TE realizes the selective extraction of the global features of the input image. In addition, we do a relu activation after each convolution.

### Decoded network structure (double decoding network)

**FIGURE 5:**
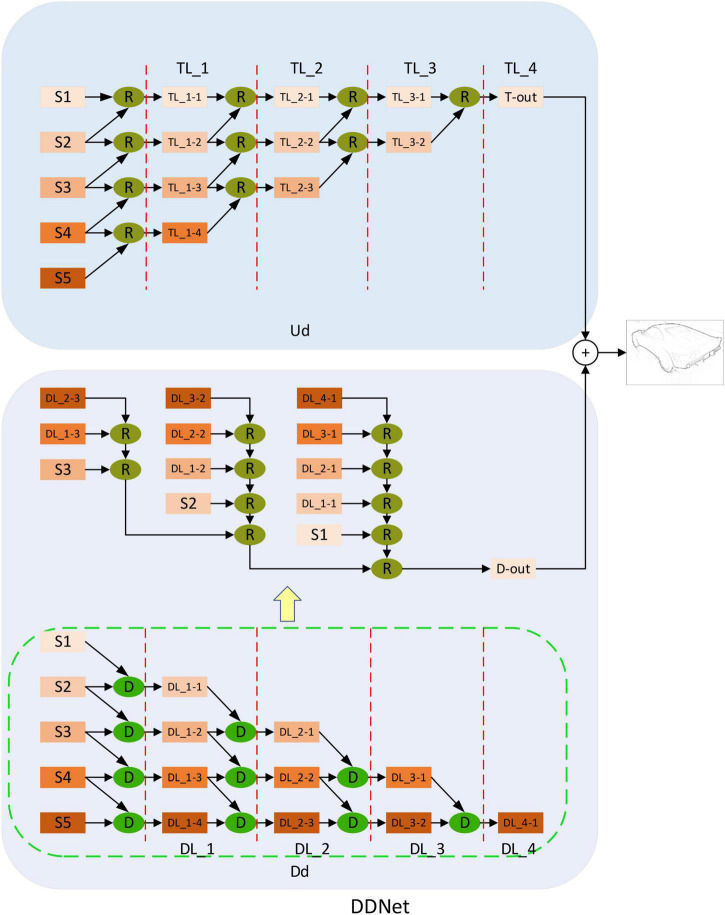
Double decoding network structure diagram. Up-sampling decoding (Ud) and down-sampling decoding (Dd) receive output from the modulation coding network. S1, S2, S3, S4, and S5 represent the five different outputs of the modulation coding network. Among them, Ud utilizes the “R” module to realize top-down feature fusion, while Dd utilizes the “D” module to realize bottom-up feature fusion. Finally, the result fusion of the two realizes full extraction and fusion of context information. TL_i (i = 1,2,3,4) represents the different stages of top-to-bottom feature fusion. The TL_1 stage takes five different outputs of the modulation coding network as inputs and obtains four different outputs (TL_1-TL_1-4) after processing by the “R” module. Then these four different outputs are used as the input of TL_2 in the next stage, and three different outputs (TL_2-1-TL_2-3) are obtained. This is processed step by step to TL_4 to obtain the output of Ud. The same DL_i (i = 1,2,3,4) symbol is similar to TL_i. It represents the different stages of top-down feature fusion. Five different outputs of the modulated coding network are taken as inputs and processed step by step by the “D” module until DL_4. In addition, the orange rectangles in different shades represent outputs of different resolutions. We detail the structure of “D” and “R” in Figure 6.

Inspired by the function of integrating feature information in IT area of biological visual pathway, we design a new decoding network – dual decoding network. Figure 5 shows the dual decoding network structure diagram proposed by us. It is different from the previous method which only uses up-sampling methods to fuse coded network output. We design two different decoding networks, which accept the same encoding network output but fuse feature information in different ways. In one of the decoding networks, the feature information of adjacent outputs is fused by up-sampling. It makes use of the “R” module (Cao et al., 2020) to up-sample the feature picture with a smaller resolution from the two adjacent feature pictures to the same size as the feature picture with a larger resolution and then merges them. In this way, a fusion output with the same size as the input is finally obtained, which is called up-sampling decoding (Ud). The other decoding network uses a down-sampling method to fuse the characteristic information of adjacent output. It uses the “D” module to down-sample the feature map with higher resolution from two adjacent feature maps to the same size as the feature map with lower resolution, and then fuse the feature map. Finally, a minimum resolution output is obtained. Then, the output of the minimum resolution is restored to the same size as the input image to obtain the final output, which is called down-sampling decoding (Dd). Finally, we fuse the output results of the two decoding networks to get the final edge. Although bottom-up fusion has achieved good results in previous methods (Liu et al., 2017; Wang et al., 2017; Lin et al., 2020), it still has the defect of insufficient context information fusion. We use the dual decoding network to fuse the bottom-up information and the top-down information, respectively, and finally combine the two to realize the full fusion of context information, and obtain good results.

**FIGURE 6:**
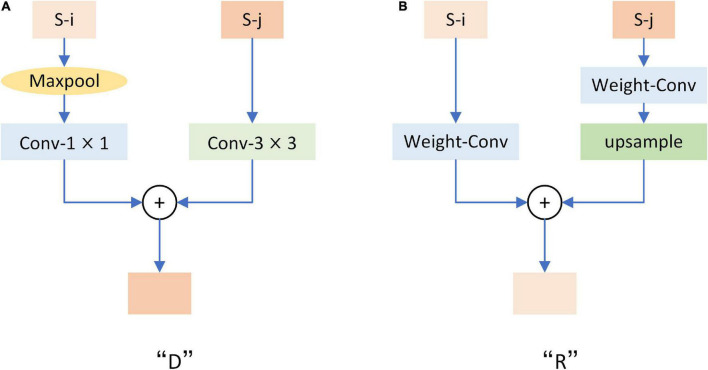
Structure diagram of “D” and “R” modules. Where S-i and S-j represent feature maps with adjacent resolution sizes. **(A)** Is the structural drawing of module “D”. **(B)** Is the structure diagram of the “R” module. Each convolution is activated by relu.

Down-sampling Module. Figure 6A shows the structure of the “D” module we used in Figure 5. The “D” module adopts a bottom-up structure to fuse feature maps with different resolutions. After “D” is entered for two adjacent feature images, the feature images with high resolution will be sampled to the same size as the feature images with low resolution. After that, the two images will be added together to achieve bottom-up fusion.

Refine Module. As shown in Figure 6B is the structure diagram of the “R” module used in Figure 5, referring to the structure proposed by Cao et al. (2020). This is the opposite of what “D” does. After input “R” for two adjacent resolution feature images, the low-resolution feature image will be sampled to the same size as the high-resolution feature image, and then the two images will be added to realize the top-down fusion.

### Loss function

To make a fair comparison with the different methods (Xie and Tu, 2015; He et al., 2019), we train with the same loss function as them. The threshold η is introduced to distinguish positive and negative sample sets in consideration of the problem of labels being tagged by multiple people. η is set to 0.2. In addition, we only calculated the loss of the final output. For a true edge graph *Y* = (*y*_*i*_, *i* = 1, …, |*Y*|), *y*_*i*_ ∈ {0, 1}, we define *Y*^+^ = {*y*_*i*_, *y*_*i*_ > η} and *Y*^−^ = {*y*_*i*_, *y*_*i*_ = 0}. *Y*^+^ and *Y*^−^ represent the positive and negative sample sets. Therefore, *l*(⋅)is calculated as follows:


(1) $$l\left(P,Y\right)=-\alpha \sum_{i\in Y^{-}}\log\left(1-p_{i}\right)-\beta \sum_{i\in Y^{+}}\log\left(p_{i}\right)$$

(2) $$\begin{array}{c} \alpha =\lambda \cdot \frac{\left|Y^{+}\right|}{\left|Y^{+}\right|+\left|Y^{-}\right|} \end{array}$$

(3) $$\begin{array}{c} \beta =\frac{\left|Y\right|}{\left|Y^{+}\right|+\left|Y^{-}\right|} \end{array}$$

In Formula (1), *P* represents the predicted contour, and *p*_*i*_ represents the value processed by a sigmoid function at pixel i. α and β are used to balance the positive and negative samples, and λ = 1.7 is the weight that controls the coefficient.

## Experiment

We evaluate the proposed approach on three public datasets: BSDS500 (Arbelaez et al., 2010), NYUDv2 (Silberman et al., 2012), and BIPED (Poma et al., 2019). These three datasets are the most widely used benchmarks in the edge detection task.

### Datasets

There are 500 images in the BSDS500. Among them, 200 training pictures, 100 verification pictures, and 200 test pictures are included. Each image is tagged by multiple people and is one of the most widely used data sets in edge detection. Using the same strategy as (Liu et al., 2017; Wang et al., 2017; Deng et al., 2018; Lin et al., 2020), we generate the amplified training set through data enhancement of BSDS500 training set. The mixed training set BSDS500-VOC was formed by mixing the amplified training set with the PASCAL VOC Context dataset (Mottaghi et al., 2014).

There are 1,449 images in the NYUD-v2. Among them, 381 training pictures, 414 verification pictures, and 654 test pictures are included. Like the previous method (Xie and Tu, 2015; Liu et al., 2017; He et al., 2019; Cao et al., 2020), we conducted training tests on RGB images and HHA features, respectively and finally averaged the outputs of RGB and HHA as the final contour output. In addition, we also adopted the same strategy as the previous method, by rotating the picture and the corresponding label four different angles (0°, 90°, 180°, 270°), and flipping the rotated picture, to realize the increase of training set.

Barcelona images for perceptual edge detection (Poma et al., 2019) contains 250 outdoor Images 1280 × 720 in size. The images were carefully annotated by experts in the field of computer vision. Same as the previous method (Poma et al., 2019), 250 images were divided into two parts, including 200 training verification images and 50 test images. In addition, we used the same strategy to increase the number of training sets by scaling the image, rotating it 15 different angles, and flipping it.

### Implementation details

We have implemented our model in PyTorch. three datasets, BSDS500 (Arbelaez et al., 2010), NYUDv2 (Silberman et al., 2012), and BIPED (Poma et al., 2019), were used to train and test the model. In the training, we use the method of transfer learning to initialize the coding network with the parameters of VGG16 trained on ImageNet (Deng et al., 2009). Other networks are initialized using a Gaussian distribution with a mean of 0 and a standard deviation of 0.01. In addition, we use the same super-parameter settings for different data sets to keep the model consistent. We used the SGD optimizer to update the parameters, setting the global learning rate to 1 × 10^–6^, momentum and weight decay to 0.9 and 2 × 10^–4^, respectively.

In order to provide a fair comparison with the previous methods, the same strategy is used to evaluate the test results as the previous methods (Xie and Tu, 2015; Liu et al., 2017; Wang et al., 2017; Deng et al., 2018). First, we apply non-maximum suppression to the test results. In the process of non-maximum suppression to the test results of BSDS500, NYUD-V2, and BIPED datasets, we set the maximum allowable errors of prediction and real annotation to 0.0075, 0.011, and 0.0075, respectively. Then, we use three widely used criteria in the field of edge detection to evaluate the result of suppression. Three commonly used evaluation criteria are optimal data set scale (ODS), optimal image scale (OIS), and average precision (AP). The F-value of each image in the dataset is calculated at a fixed threshold and its average value is computed. Then calculate the maximum value of all average values under different thresholds, which is the best data set scale ODS. Calculate the best F value of each image under different thresholds, and then calculate the average of all F values, which is the best image scale OIS. AP is the average accuracy within a given threshold range of (0–1.0) (Martin et al., 2004). In addition, under different threshold conditions, the accuracy P and regression R of the whole dataset can be described as PR curves.

P is calculated as follows:


(4) $$\text{P}=\frac{\text{TP}}{\left(\mathrm{TP}+\mathrm{FP}\right)}$$

TP and FP represent the correct number and false number of contour pixels.

R is calculated as follows:


(5) $$\text{R}=\frac{\text{TP}}{\left(\mathrm{TP}+\mathrm{FN}\right)}$$

TP and FN represent the correct number and missed number of contour pixels.

F value is calculated as follows:


(6) $$\text{F}=\frac{\left(\text{P}\;\times \;\text{R}\right)}{\left[\left(1-\alpha \right)P+\alpha \text{R}\right]}$$

α is the weight, generally 0.5. P and R stand for accuracy and regression, respectively.

### Ablation study

**FIGURE 7:**
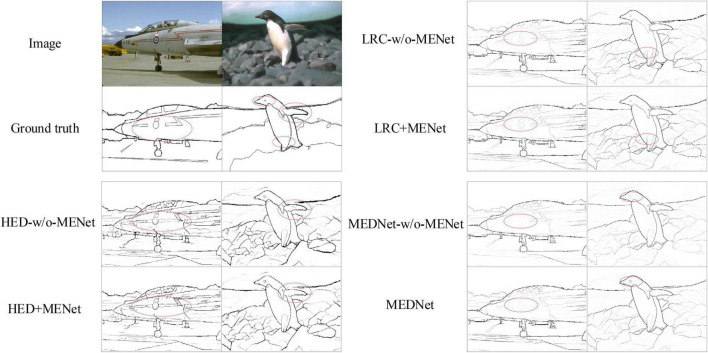
Schematic plots of the output of Holistically-nested edge detection (HED), Richer convolutional features (RCF), LRC and modulation encoding and decoding network (MEDNet) before and after combining with modulation-enhancement network (MENet). On the left, from top to bottom, are the original image, the ground truth, HED-w/o-MENet and HED + MENet. The right part runs from top to bottom for LRC-w/o-MENet, LRC + MENet, and MEDNet. Source photos: BSDS500 dataset - publicly available dataset.

In order to further illustrate the effectiveness of our proposed method in this paper, we conducted a detailed experimental analysis and evaluation of the proposed MENet on the BSDS500 dataset. First, without changing other conditions, we combined MENet with some of the previous methods, such as combining MENet with HED (Xie and Tu, 2015), RCF (Liu et al., 2017), and LRC (Lin et al., 2020), respectively. Then we compared the results of these models before and after combining MENet. The experimental comparison results are shown in Table 1. Among them, HED’s experiments used enhanced BSDS500 data sets and only tested single-scale results. The results of the other models all used a mixed dataset BSDS500-VOC and tested multi-scale results. As you can see from the results in Table 1, the MENet we propose in this paper improves the ODS of HED by 0.3%, which is 0.1% higher than the enhanced data set for HED. The ODS of RCF increased by 0.1% and the OIS by 0.3%. LRC’s results also improved by 0.1%. In addition, it can be seen from the results in the table that the OIS of the model combined with MENet also improved to varying degrees. This also proves the effectiveness of MENet proposed by us, which can effectively improve the feature extraction ability of the coding network and improve the overall performance of the model. In addition, Figure 7 shows the results of different models and MEDNet before and after combining MENet. From figure, we can also see that combining MENet reduces the extracted texture and useless information, and increases the required detailed information.

**TABLE 1:** Comparison of results of other models before and after combining modulation-enhancement network (MENet) on BSDS500 data set.

Method	MENet		ODS	OIS	AP
HED (Xie and Tu, 2015)	×	SS	0.788	0.808	0.840
	√		**0.791 (↑0.003)**	**0.811 (↑0.003)**	0.809
RCF (Liu et al., 2017)	×	MS	0.811	0.830	–
	√		**0.812 (↑0.001)**	**0.833 (↑0.003)**	**0.871**
LRC (Lin et al., 2020)	×	MS	0.816	0.836	0.864
	√		**0.817 (↑0.001)**	**0.839 (↑0.003)**	**0.867 (↑0.003)**

“×” means without MENet and “√” means with MENet. SS, single-scale results; MS, multi-scale results. Except for HED (Xie and Tu, 2015), the results are on BSDS500-VOC. “↑”indicates improved results.

In order to further verify the consistency of MENet under different conditions. In addition to the above methods to verify the effectiveness of MENet, we also tested the results of MENet on BSDS500-VOC and BSDS500 by self-separation test. The experimental results are shown in Table 2. Experimental results show that MENet improves the performance of the model in both cases, which proves that MENet has consistent performance in different cases.

**TABLE 2:** Comparison of results of modulation encoding and decoding network (MEDNet) under different conditions.

Method		ODS	OIS	AP
MEDNet	SS	**0.811**	**0.831**	**0.849**
MEDNet-w/o-MENet	SS	0.807 **(↓0.004)**	0.828 **(↓0.003)**	0.842 **(↓0.007)**
MEDNet	MS	**0.825**	**0.845**	**0.872**
MEDNet-w/o-MENet	MS	0.822 **(↓0.003)**	0.844 **(↓0.001)**	0.857 **(↓0.015)**
MEDNet-w/o-VOC	SS	**0.797**	**0.820**	0.812
MEDNet-w/o-MENet-w/o-VOC	SS	0.796 **(↓0.001)**	0.820	0.822 **(↑0.010)**
MEDNet-w/o-VOC	MS	**0.811**	**0.833**	0.831
MEDNet-w/o-MENet-w/o-VOC	MS	0.809 **(↓0.002)**	0.832 **(↓0.001)**	0.836 **(↑0.005)**
**Comparison of the results of different decoding networks**				
MCNet + DDNet	MS	**0.825**	**0.845**	**0.872**
MCNet + Dd	MS	0.823	0.844	0.865
MCNet + Ud	MS	0.821	0.844	0.870

MEDNet-w/o-MENet means no MENet, and MEDNet-w/o-VOC means no mixed data set. “↓” Indicates a decrease in the result.

We then validate the proposed dual decoding network on the BSDS500 hybrid dataset. We combine the dual decoding network DDNet, the up-sampling decoding network Ud, and the down-sampling decoding network Dd with the modulation coding network MCNet, respectively. Denoted as MCNet + DDNet, MCNet + Dd, MCNet + Ud. We test their experimental results, and the results are shown in Table 2. Under other conditions being the same, the multi-scale ODS of the dual decoding network DDNet is 0.4% and 0.2% higher than the up-sampling decoding Ud and the down-sampling decoding Dd, respectively. In addition, it is worth noting that the down-sampling decoding Dd is 0.2% higher than the up-sampling decoding Ud, which further proves the effectiveness of our proposed down-sampling decoding and dual decoding networks in this paper.

### Comparison with other works

BSDS500. We trained our model on the BSDS500-VOC hybrid training set and conducted a detailed experimental analysis and evaluation of the test results. We compare the final results with the previous edge detection methods. They include traditional edge detection methods, biomimetic vision edge detection methods, edge detection methods based on unsupervised learning, and edge detection method based on CNN. Such as Canny (1986), SCO (Yang et al., 2015a), SED (Akbarinia and Parraga, 2018), gPb (Arbelaez et al., 2010), OEF (Hallman and Fowlkes, 2015), SE (Dollár and Zitnick, 2014), DeepContour (Shen et al., 2015), DeepEdge (Bertasius et al., 2015), COB (Maninis et al., 2016), HED (Xie and Tu, 2015), RCF (Liu et al., 2017), CED (Wang et al., 2017), LPCB (Deng et al., 2018), DRC (Cao et al., 2020), LRC (Lin et al., 2020), DSCD (Deng and Liu, 2020), BDCN (He et al., 2019), PIDiNet (Su et al., 2021), EDTER (Pu et al., 2022). Table 3 shows the quantitative comparison results between the proposed method and other methods.

**TABLE 3:** Quantitative comparison results between the proposed method and other methods on the BSDS500 test set.

Method	ODS	OIS	AP	P(M)	FLOPs (G)
Human (Martin et al., 2004)	0.803	0.803	–	–	–
Canny (Canny, 1986)	0.611	0.676	0.520	–	–
SCO (Yang et al., 2015a)	0.670	0.710	0.710	–	–
SED (Akbarinia and Parraga, 2018)	0.710	0.740	0.740	–	–
gPb (Arbelaez et al., 2010)	0.729	0.755	0.745	–	–
OEF (Hallman and Fowlkes, 2015)	0.746	0.770	0.815	–	–
SE (Dollár and Zitnick, 2014)	0.743	0.764	0.800	–	–
DeepContour (Shen et al., 2015)	0.757	0.776	0.790	–	–
DeepEdge (Bertasius et al., 2015)	0.753	0.772	0.787	–	–
COB (Maninis et al., 2016)	0.793	0.819	0.849	28.8^†^	–
HED (Xie and Tu, 2015)	0.788	0.808	0.840	14.7^‡^	93.2^‡^
RCF-SS-VOC (Liu et al., 2017)	0.806	0.823	0.839	14.8^‡^	79.7^‡^
RCF-MS-VOC (Liu et al., 2017)	0.811	0.830	0.846		
CED-SS (Wang et al., 2017)	0.803	0.820	0.871	21.4^†^	138.8^†^
CED-MS-VOC (Wang et al., 2017)	0.815	0.833	0.889		
LPCB-SS-VOC (Deng et al., 2018)	0.808	0.824	–	15.7^†^	121.5^†^
LPCB-MS-VOC (Deng et al., 2018)	0.815	0.834	0.827		
DRC-SS-VOC (Cao et al., 2020)	0.802	0.818	0.800	17.7^‡^	124.2^‡^
DRC-MS-VOC (Cao et al., 2020)	0.817	0.832	0.836		
LRC-SS-VOC (Lin et al., 2020)	0.802	0.821	0.830	24.8^‡^	174.4^‡^
LRC-MS-VOC (Lin et al., 2020)	0.816	0.836	0.864		
DSCD-SS-VOC (Deng and Liu, 2020)	0.813	0.836	0.847	34.07^†^	135.3^†^
DSCD-MS-VOC (Deng and Liu, 2020)	0.822	**0.859**	0.863		
BDCN-SS-VOC (He et al., 2019)	0.820	0.838	0.888	16.3^‡^	95.1^‡^
BDCN-MS-VOC (He et al., 2019)	0.828	0.844	**0.890**		
PiDiNet-SS-VOC (Su et al., 2021)	0.807	0.823	–	0.71^‡^	16.6^‡^
EDTER-SS-VOC (Pu et al., 2022)	**0.832**	0.847	0.886	–	332.0^†^
EDTER-MS-VOC (Pu et al., 2022)	**0.848**	**0.865**	**0.903**	–	
MEDNet-SS-VOC	0.811	0.831	0.849	55.2^‡^	179.5^‡^
MEDNet-MS-VOC	**0.825**	**0.845**	**0.872**		

VOC, mixed data set BSDS500-VOC; SS, single-scale results; MS, multi-scale results. The best two results are marked with red and blue, respectively. ^‡^Stands for the result of our test. ^†^Indicates the result of the reference. FLOPs are calculated based on a 320 × 320 image.

**FIGURE 8:**
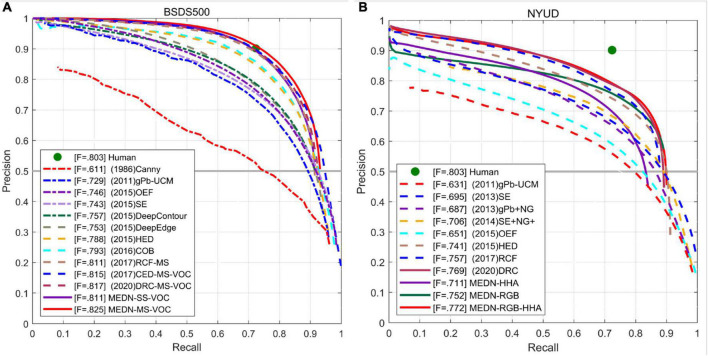
PR curves of the proposed method and other methods on BSDS500 **(A)** and NYUD-v2 **(B)** data sets.

As can be seen from Table 3, the Transformer based method achieves the best results among all methods. However, EDTER also has the highest floating-point operations per second (FLOPs). EDTER (Pu et al., 2022) brings 332.0G FLOPs in Stage I and 470.25G FLOPs in Stage II, which is much higher than other methods. Among all CNN based methods, our method achieves the top three results. Multi-scale ODS = 0.825, single-scale ODS = 0.811. Our multiscale results are 0.3% lower than the best BDCN, but our OIS exceeds BDCN by 0.1%. Compared with other methods, the ODS of our method is 0.3% higher than that of DSCD multi-scale ODS. It is 0.8% higher than DRC multi-scale ODS and 0.9% higher than DRC single-scale ODS. Moreover, the single-scale ODS of our method is the same as the multi-scale ODS of RCF, which further proves that our model has strong competitiveness. Figure 8A shows the PR curve of our method and other methods.

NYUD-V2. We performed an experimental evaluation of our model on the NYUD-V2 dataset. As before, we trained our model on RGB image and HHA feature map, and then tested them separately. Finally, we got three outputs: RGB, HHA, RGB-HHA. Where RGB-HHA is the average output of RGB and HHA. We compare the three output results with those from other methods. Such as gPb-UCM (Arbelaez et al., 2010), SE (Dollár and Zitnick, 2014), gPb + NG (Gupta et al., 2013), SE + NG + (Gupta et al., 2014), OEF (Hallman and Fowlkes, 2015), HED (Xie and Tu, 2015), RCF (Liu et al., 2017), LPCB (Deng et al., 2018), DRC (Cao et al., 2020), LRC (Lin et al., 2020), BDCN (He et al., 2019), AMH (Xu et al., 2018), PiDiNet (Su et al., 2021), EDTER (Pu et al., 2022). Note that, AMH-Net applies the deeper ResNet50 to construct the edge detector. The experimental results are shown in Table 4.

**TABLE 4:** Quantitative comparison results between the proposed method and other methods on nyud-v2 test set.

Method	Input	ODS	OIS	AP
gPb-UCM (Arbelaez et al., 2010)	RGB	0.631	0.661	0.562
SE (Dollár and Zitnick, 2014)		0.695	0.708	0.719
gPb + NG (Gupta et al., 2013)		0.687	0.716	0.629
SE + NG + (Gupta et al., 2014)		0.706	0.734	0.549
OEF (Hallman and Fowlkes, 2015)		0.651	0.667	0.653
HED (Xie and Tu, 2015)	RGB	0.717	0.732	0.704
	HHA	0.681	0.695	0.674
	RGB-HHA	0.741	0.757	0.749
RCF (Liu et al., 2017)	RGB	0.729	0.742	0.693
	HHA	0.705	0.715	0.650
	RGB-HHA	0.757	0.771	0.749
LPCB (Deng et al., 2018)	RGB	0.739	0.754	–
	HHA	0.707	0.719	–
	RGB-HHA	0.762	0.778	–
DRC (Cao et al., 2020)	RGB	0.749	0.762	0.718
	HHA	**0.711**	0.722	0.677
	RGB-HHA	0.769	0.782	0.771
LRC (Lin et al., 2020)	RGB	0.737	0.750	0.686
	HHA	0.697	0.708	0.642
	RGB-HHA	0.759	0.771	0.748
BDCN (He et al., 2019)	RGB	0.748	0.763	0.770
	HHA	0.707	0.719	**0.731**
	RGB-HHA	0.765	0.781	**0.813**
AMH-Net-ResNet50 (Xu et al., 2018)	RGB	0.744	0.758	**0.765**
	HHA	**0.716**	**0.729**	**0.734**
	RGB-HHA	**0.771**	**0.786**	**0.802**
PiDiNet (Su et al., 2021)	RGB-HHA	0.756	0.773	–
EDTER (Pu et al., 2022)	RGB	**0.774**	**0.789**	**0.797**
MEDNet	RGB	**0.752**	**0.766**	0.723
	HHA	**0.711**	**0.723**	0.681
	RGB-HHA	**0.772**	**0.787**	**0.776**

The best two results are marked with red and blue, respectively.

**FIGURE 9:**
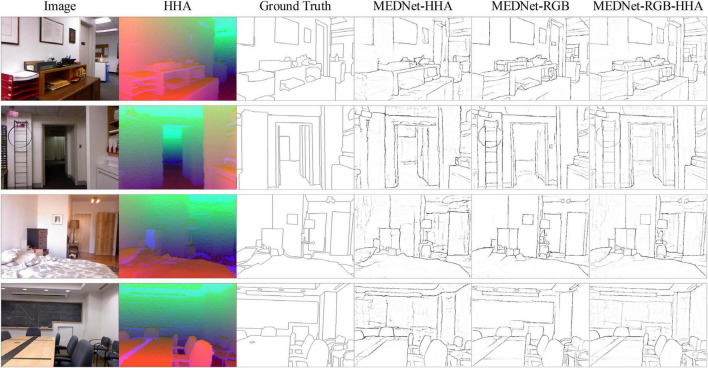
Partial test results of the proposed method on NYUD-V2 data set are presented. Source photos: BSDS500 dataset - publicly available dataset.

As can be seen from Table 4, our method achieves the best performance at present, ODS = 0.772. And in the edge detection method based on VGG16, the ODS and OIS of the three outputs of our model are better than the current best method BDCN. Among them, RGB-HHA increased by 0.7%, RGB increased by 0.8% and HHA increased by 0.4%. Our proposed method also achieves the best RGB-HHA, RGB, compared with AMH based on a deeper ResNet50 model. It shows that our method shows consistent performance in different data sets and has strong competitiveness compared with other methods. Figure 9 is part of the output result graph randomly selected by us. Figure 8B shows the PR curves of the proposed method compared with other methods.

Barcelona images for perceptual edge detection. We train and test our proposed model on BIPED, a carefully annotated and publicly available edge dataset presented in Poma et al. (2019). Poma et al. (2019) tested some of the previous methods using BIPED and recorded the results. Combined with their records, we compared the test results with the results of other methods on the BIPED dataset. Including SED (Akbarinia and Parraga, 2018), HED (Xie and Tu, 2015), CED (Wang et al., 2017), RCF (Liu et al., 2017), BDCN (He et al., 2019), DexiNed (Poma et al., 2019). Table 5 shows the comparison results between our method and other methods.

**TABLE 5:** Quantitative comparison results between the proposed method and other methods on barcelona images for perceptual edge detection (BIPED) test sets.

Method	ODS	OIS	AP
SED (Akbarinia and Parraga, 2018)	0.717	0.731	0.756
HED (Xie and Tu, 2015)	0.829	0.847	0.869
CED (Wang et al., 2017)	0.795	0.815	0.830
RCF (Liu et al., 2017)	0.843	0.859	0.882
BDCN (He et al., 2019)	0.839	0.854	0.887
DexiNed (Poma et al., 2019)	**0.859**	**0.867**	**0.905**
MEDNet-SS	**0.896**	**0.900**	**0.920**

SS, single-scale result. The best two results are marked with red and blue, respectively.

**FIGURE 10:**
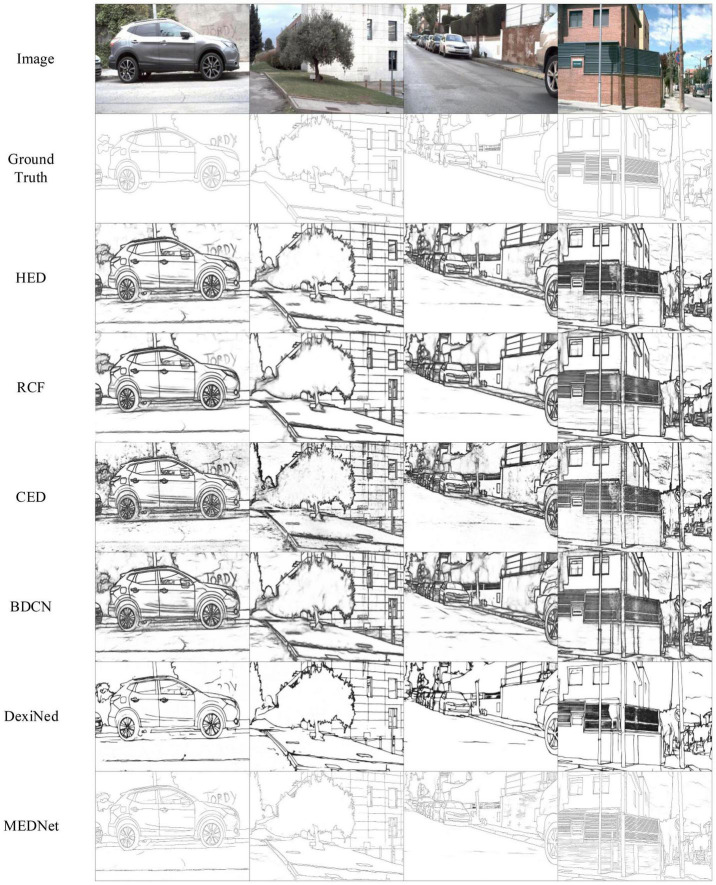
Comparison of results of the proposed method with other methods on barcelona images for perceptual edge detection (BIPED) datasets. Among them, in order to make a fair comparison, the results of other methods are the same as those used in Poma et al. (2019). Source photos: BSDS500 dataset - publicly available dataset.

The results in Table 5 show that our method achieves the best performance among the current methods, with ODS = 0.896. That’s a 3.7% improvement over the ODS of the current best DexiNed. It is 5.7% higher than BDCN. And our proposed methods OIS and AP also achieved the best results. This not only shows that our method performs consistently on different data sets but also proves that our method is more competitive than other methods. As shown in Figure 10, the output of our method is compared with that of other methods. These outputs are the result of non-maximum suppression. It can be seen that our method has less background texture and finer edges than other methods.

## Conclusion

Inspired by the selective mechanism of V1, V2, and V4 in the biological visual pathway, and combined with the connection between CNN and biological vision, we design a MENet using the self-attention mechanism in the transformer. The modulation and enhancement of the coding network are realized by combining MENet with the coding network so that the structure and function of the modulation-enhanced coding network MCNet and the biological visual pathway form a more reasonable correspondence. In addition, in order to fully fuse the context information output by the encoding network, we innovatively propose a dual decoding network in this paper. One decoding network integrates bottom-up feature information, and the other one integrates top-down information. The two do not interfere with each other during the fusion process. Finally, we fuse the output of the two decoding networks to get the final output. We train and test the new edge detection model MEDNet, which is combined with MENet, encoding network, and DDNet, on several data sets. The results show that our method achieves good performance on several data sets and has strong competitiveness compared with other methods. In addition, inspired by the selective mechanism of V1, V2, and V4 in biological vision, we combined them with CNN to design a new edge detection method. It changes the current approach of using only CNN or biological vision. This provides a new idea for future research on edge detection. Other effective physiological mechanisms in the biological vision for edge detection tasks can be combined with the current CNN-based method with good performance.

## Data availability statement

The datasets presented in this study can be found in online repositories. The names of the repository/repositories and accession number(s) can be found below: https://github.com/zwuser1227/MEDNet-Edge-detection.

## Author contributions

CL was responsible for manuscript preparation and worked as a supervisor for all procedures. ZZ was responsible for programming and data processing. YQ participated in discussions and revisions. YP participated in discussions and revisions. All authors contributed to the article and approved the submitted version.

## References

[B1] Akbarinia A. Parraga C. A. (2018). Feedback and surround modulated boundary detection. *International Journal of Computer Vision* 126 1367–1380. 10.1007/s11263-017-1035-5

[B2] Allen H. A. Humphreys G. W. Colin J. Neumann H. (2018). Ventral extra-striate cortical areas are required for human visual texture segmentation. *Journal of Vision* 9 1–14. 10.1167/9.9.2 19761335

[B3] Arbelaez P. Maire M. Fowlkes C. Malik J. (2010). Contour detection and hierarchical image segmentation. *IEEE transactions on pattern analysis and machine intelligence* 33 898–916. 10.1109/TPAMI.2010.161 20733228

[B4] Bear M. Connors B. Paradiso M. A. (2020). *Neuroscience: Exploring the Brain, Enhanced 4th Edition: Exploring the Brain.* Burlington, MA: Jones & Bartlett Learning.

[B5] Bertasius G. Shi J. Torresani L. (2015). “Deepedge: A multi-scale bifurcated deep network for top-down contour detection,” in *Proceedings of the IEEE conference on computer vision and pattern recognition*, (Boston, MA), 4380–4389. 10.1109/CVPR.2015.7299067

[B6] Canny J. (1986). A computational approach to edge detection. *IEEE Transactions on pattern analysis and machine intelligence* 8 679–698. 21869365

[B7] Cao Y.-J. Lin C. Li Y.-J. (2020). Learning Crisp Boundaries Using Deep Refinement Network and Adaptive Weighting Loss. *IEEE Transactions on Multimedia* 23 761–771. 10.1109/TMM.2020.2987685

[B8] Deng J. Dong W. Socher R. Li L.-J. Li K. Fei-Fei L. (2009). “Imagenet: A large-scale hierarchical image database,” in *Proceedings of the 2009 IEEE conference on computer vision and pattern recognition*, (Miami, FL: IEEE), 248–255. 10.1109/CVPR.2009.5206848

[B9] Deng R. Liu S. (2020). “Deep Structural Contour Detection,” in *Proceedings of the 28th ACM International Conference on Multimedia*, 304–312. 10.1145/3394171.3413750

[B10] Deng R. Liu S. Wang J. Wang H. Zhao H. Zhang X. (2021). “Learning to Decode Contextual Information for Efficient Contour Detection,” in *Proceedings of the 29th ACM International Conference on Multimedia*, 4435–4443. .

[B11] Deng R. Shen C. Liu S. Wang H. Liu X. (2018). “Learning to predict crisp boundaries,” in *Proceedings of the European Conference on Computer Vision (ECCV)*, 562–578. . 10.1007/978-3-030-01231-1_35

[B12] Dollár P. Zitnick C. L. (2014). Fast edge detection using structured forests. *IEEE transactions on pattern analysis and machine intelligence* 37 1558–1570. 10.1109/TPAMI.2014.2377715 26352995

[B13] Dosovitskiy A. Beyer L. Kolesnikov A. Weissenborn D. Houlsby N. (2020). *An Image is Worth 16x16 Words: Transformers for Image Recognition at Scale. .*

[B14] Duda R. O. Hart P. E. (2003). Pattern classification and scene analysis. *IEEE Transactions on Automatic Control* 19 462–463.

[B15] Ferrari V. Fevrier L. Jurie F. Schmid C. (2007). Groups of Adjacent Contour Segments for Object Detection. *IEEE Transactions on Pattern Analysis and Machine Intelligence* 30 36–51. 10.1109/TPAMI.2007.1144 18000323

[B16] Fukushima K. Miyake S. Ito T. (1988). Neocognitron: A Self-Organizing Neural Network Model for a Mechanism of Visual Pattern Recognition. *IEEE Transactions on Systems Man and Cybernetics SMC-* 13 826–834. 10.1109/TSMC.1983.6313076

[B17] Grigorescu C. Petkov N. Westenberg M. A. (2003). Contour detection based on nonclassical receptive field inhibition. *IEEE Transactions on image processing* 12 729–739. 10.1109/TIP.2003.814250 18237948

[B18] Gross C. G. Rocha-Miranda C. E. Bender D. B. (1972). Visual properties of neurons in inferotemporal cortex of the macaque. *Journal of neurophysiology* 35 96–111. 10.1152/jn.1972.35.1.96 4621506

[B19] Gupta S. Arbelaez P. Malik J. (2013). “Perceptual organization and recognition of indoor scenes from RGB-D images,” in *Proceedings of the IEEE conference on computer vision and pattern recognition*, 564–571. . 10.1109/CVPR.2013.79

[B20] Gupta S. Girshick R. Arbeláez P. Malik J. (2014). “Learning rich features from RGB-D images for object detection and segmentation,” in *European conference on computer vision*, eds Fleet D. Pajdla T. Schiele B. Tuytelaars T. (Cham: Springer), 345–360. 10.1007/978-3-319-10584-0_23

[B21] Hallman S. Fowlkes C. C. (2015). “Oriented edge forests for boundary detection,” in *Proceedings of the IEEE conference on computer vision and pattern recognition*, (Boston, MA), 1732–1740. 10.1109/CVPR.2015.7298782

[B22] Hao W. Andolina I. M. Wang W. Zhang Z. (2021). Biologically inspired visual computing: the state of the art. *Frontiers of Computer Science* 15:151304. 10.1007/s11704-020-9001-8

[B23] He J. Zhang S. Yang M. Shan Y. Huang T. (2019). “Bi-directional cascade network for perceptual edge detection,” in *Proceedings of the IEEE/CVF Conference on Computer Vision and Pattern Recognition*, (Piscataway, NJ: IEEE), 3828–3837. 10.1109/CVPR.2019.00395

[B24] He K. Zhang X. Ren S. Sun J. (2016). “Deep Residual Learning for Image,” in *Proceedings of the 2016 IEEE Conference on Computer Vision and Pattern Recognition (CVPR*, (Las Vegas, NV). 10.1109/CVPR.2016.90

[B25] Hubel D. H. Wiesel T. N. (1962). Receptive fields, binocular interaction and functional architecture in the cat’s visual cortex. *The Journal of physiology* 160 106–154. 10.1113/jphysiol.1962.sp006837 14449617 PMC1359523

[B26] Lin C. Cui L. Li F. Cao Y. (2020). Lateral refinement network for contour detection. *Neurocomputing* 409 361–371. 10.1016/j.neucom.2020.06.069

[B27] Lin G. Milan A. Shen C. Reid I. (2017). “Refinenet: Multi-path refinement networks for high-resolution semantic segmentation,” in *Proceedings of the IEEE conference on computer vision and pattern recognition*, (Honolulu, HI), 1925–1934. 10.1109/CVPR.2017.549

[B28] Liu L. Ouyang W. Wang X. Fieguth P. Chen J. Liu X. (2020). Deep Learning for Generic Object Detection: A Survey. *Int J Comput Vision* 128 261–318. 10.1007/s11263-019-01247-4

[B29] Liu Y. Cheng M.-M. Hu X. Wang K. Bai X. (2017). “Richer convolutional features for edge detection,” in *Proceedings of the IEEE conference on computer vision and pattern recognition*, (Piscataway, NJ: IEEE), 3000–3009. 10.1109/CVPR.2017.622

[B30] Liu Z. Lin Y. Cao Y. Hu H. Wei Y. Zhang Z. (2021). Swin Transformer: Hierarchical Vision Transformer using Shifted Windows. *arXiv preprintarXiv:2103.14030.* 10.1109/ICCV48922.2021.00986

[B31] Long J. Shelhamer E. Darrell T. (2015). “Fully convolutional networks for semantic segmentation,” in *Proceedings of the IEEE conference on computer vision and pattern recognition*, (Boston, MA), 3431–3440. 10.1109/CVPR.2015.7298965 27244717

[B32] Luck S. J. Leonardo C. Hillyard S. A. Robert D. (1997). Neural mechanisms of spatial selective attention in areas V1, V2, and V4 of macaque visual cortex. *Journal of Neurophysiology* 77 24–42. 10.1152/jn.1997.77.1.24 9120566

[B33] Maninis K.-K. Pont-Tuset J. Arbeláez P. Van Gool L. (2016). *Convolutional oriented boundaries, European conference on computer vision.* Cham: Springer, 580–596. 10.1007/978-3-319-46448-0_35

[B34] Marcus D. S. Essen D. (2002). Scene Segmentation and Attention in Primate Cortical Areas V1 and V2. *Journal of Neurophysiology* 88 2648–2658. 10.1152/jn.00916.2001 12424300

[B35] Martin D. R. Fowlkes C. C. Malik J. (2004). Learning to detect natural image boundaries using local brightness, color, and texture cues. *IEEE transactions on pattern analysis and machine intelligence* 26 530–549. 10.1109/TPAMI.2004.1273918 15460277

[B36] Mishkin M. Ungerleider L. G. Macko K. A. (1983). Object vision and spatial vision: two cortical pathways. *Trends in neurosciences* 6 414–417.

[B37] Mottaghi R. Chen X. Liu X. Cho N.-G. Lee S.-W. Fidler S. Urtasun R. Yuille A. (2014). The role of context for object detection and semantic segmentation in the wild, Proceedings of the IEEE conference on computer vision and pattern recognition, pp. 891-898.Columbus, OH 10.1109/CVPR.2014.119

[B38] Muthukrishnan R. Radha M. (2012). edge detection techniques for image segmentation. *International Journal of Computer ence & Information Technolo* 3 250–254. 10.5121/ijcsit.2011.3620

[B39] Okazawa G. Tajima S. Komatsu H. (2016). Gradual development of visual texture-selective properties between macaque areas V2 and V4. *Cerebral Cortex* 27 4867–4880. 10.1093/cercor/bhw282 27655929

[B40] Poma X. S. Riba E. Sappa A. D. (2019). “Dense Extreme Inception Network: Towards a Robust CNN Model for Edge Detection,” in *Proceedings of the IEEE/CVF Winter Conference on Applications of Computer Vision*, (Snowmass Village, CO: IEEE).

[B41] Prewitt J. M. (1970). Object enhancement and extraction. *Picture processing and Psychopictorics* 10 15–19.

[B42] Pu M. Huang Y. Liu Y. Guan Q. Ling H. (2022). “EDTER: Edge Detection with Transformer,” in *Proceedings of the IEEE/CVF Conference on Computer Vision and Pattern Recognition*, (New Orleans, LA), 1402–1412. 10.1109/CVPR52688.2022.00146

[B43] Shen W. Wang X. Wang Y. Bai X. Zhang Z. (2015). “Deepcontour: A deep convolutional feature learned by positive-sharing loss for contour detection,” in *Proceedings of the IEEE conference on computer vision and pattern recognition*, (Boston, MA), 3982–3991.

[B44] Silberman N. Hoiem D. Kohli P. Fergus R. (2012). *Indoor segmentation and support inference from rgbd images, European conference on computer vision.* Cham: Springer, 746–760. 10.1007/978-3-642-33715-4_54

[B45] Simonyan K. Zisserman A. (2015). Very deep convolutional networks for large-scale image recognition. *arXiv:1409.1556.*

[B46] Su Z. Liu W. Yu Z. Hu D. Liao Q. Tian Q. (2021). “Pixel Difference Networks for Efficient Edge Detection,” in *Proceedings of the IEEE/CVF International Conference on Computer Vision*, 5117–5127. . 10.1109/ICCV48922.2021.00507

[B47] Tanaka K. (1996). Inferotemporal cortex and object vision. *Annual review of neuroscience* 19 109–139. 10.1146/annurev.ne.19.030196.000545 8833438

[B48] Tang Q. Sang N. Liu H. (2019). Learning Nonclassical Receptive Field Modulation for Contour Detection. *IEEE Transactions on Image Processing* 29 1192–1203. 10.1109/TIP.2019.2940690 31536000

[B49] Ungerleider L. G. Haxby J. V. (1994). ‘What’and ‘where’in the human brain. *Current opinion in neurobiology* 4 157–165. 10.1016/0959-4388(94)90066-3 8038571

[B50] Wang W. Xie E. Li X. Fan D. P. Shao L. (2021). Pyramid Vision Transformer: A Versatile Backbone for Dense Prediction without Convolutions. *arXiv preprint arXiv:2102.12122.* 10.1109/ICCV48922.2021.00061

[B51] Wang Y. Zhao X. Huang K. (2017). “Deep crisp boundaries,” in *Proceedings of the IEEE Conference on Computer Vision and Pattern Recognition*, 3892–3900. c^**^. 10.1109/CVPR.2017.187

[B52] Wibisono J. K. Hang H.-M. (2020a). Fined: Fast inference network for edge detection. *arXiv preprint arXiv:2012.08392.* 10.1109/ICME51207.2021.9428230

[B53] Wibisono J. K. Hang H.-M. (2020b). “Traditional method inspired deep neural network for edge detection,” in *proceedings of the 2020 IEEE International Conference on Image Processing (ICIP)*, (Piscataway, NJ: IEEE), 678–682. 10.1109/ICIP40778.2020.9190982

[B54] Xie S. Tu Z. (2015). “Holistically-nested edge detection,” in *Proceedings of the IEEE international conference on computer vision*, (Santiago), 1395–1403. 10.1109/ICCV.2015.164

[B55] Xu D. Ouyang W. Alameda-Pineda X. Ricci E. Sebe N. (2018). *Learning Deep Structured Multi-Scale Features using Attention-Gated CRFs for Contour Prediction. .*

[B56] Yang J. Price B. Cohen S. Lee H. Yang M.-H. (2016). “Object contour detection with a fully convolutional encoder-decoder network,” in *IEEE Conference on Computer Vision and Pattern Recognition* (Las Vegas, NV: IEEE), 193–202.

[B57] Yang K. F. Gao S. B. Li Y. J. (2015b). “Efficient illuminant estimation for color constancy using grey pixels,” in *Proceedings of the 2015 IEEE Conference on Computer Vision and Pattern Recognition (CVPR). .* 10.1109/CVPR.2015.7298838

[B58] Yang K. F. Gao S. B. Guo C. F. Li C. Y. Li Y. J. (2015a). Boundary Detection Using Double-Opponency and Spatial Sparseness Constraint. *IEEE Transactions on Image Processing A Publication of the IEEE Signal Processing Society* 24 2565. 10.1109/TIP.2015.2425538 25910090

[B59] Yoshioka T. Dow B. M. Vautin R. G. (1996). Neuronal mechanisms of color categorization in areas V1. *V2 and V4 of macaque monkey visual cortex. Behavioural Brain Research* 76 51–70. 10.1016/0166-4328(95)00183-2 8734043

